# Effects of Anesthesia on Ozone-Induced Lung and Systemic Inflammation

**DOI:** 10.1007/s00408-022-00514-5

**Published:** 2022-02-23

**Authors:** Miranda L. Wilson, Jarl A. Thysell, Kristen K. Baumann, Danny V. Quaranta, W. Sandy Liang, Michelle A. Erickson

**Affiliations:** 1 Veterans Administration Puget Sound Healthcare System, 1660 S. Columbian Way, S-182, Seattle, WA 98108 USA; 2 Division of Gerontology and Geriatric Medicine, Department of Medicine, University of Washington, 325 9th Avenue, Box 359755, Seattle, WA 98104 USA; 3 Present Address: Department of Cell, Developmental, and Regenerative Biology, Icahn School of Medicine at Mount Sinai, 1 Gustave L. Levy Pl, New York, NY 10029 USA

**Keywords:** Ozone, Inflammation, Isoflurane, Ketamine, Xylazine, Atipamezole, Serum amyloid A

## Abstract

**Purpose:**

Anesthetics are required for procedures that deliver drugs/biologics, infectious/inflammatory agents, and toxicants directly to the lungs. However, the possible confounding effects of anesthesia on lung inflammation and injury are underreported. Here, we evaluated the effects of two commonly used anesthetic regimens on lung inflammatory responses to ozone in mice.

**Methods:**

We tested the effects of brief isoflurane (Iso) or ketamine/xylazine/atipamezole (K/X/A) anesthesia prior to ozone exposure (4 h, 3 ppm) on lung inflammatory responses in mice. Anesthesia regimens modeled those used for non-surgical intratracheal instillations and were administered 1–2 h or 24 h prior to initiating ozone exposure.

**Results:**

We found that Iso given 1–2 h prior to ozone inhibited inflammatory responses in the lung, and this effect was absent when Iso was given 23–24 h prior to ozone. In contrast, K/X/A given 1–2 h prior to ozone increased lung and systemic inflammation.

**Conclusion:**

Our results highlight the need to comprehensively evaluate anesthesia as an experimental variable in the assessment of lung inflammation in response to ozone and other inflammatory stimuli.

## Introduction

Ozone (O_3_) is a widespread air toxicant with well-established inflammatory effects in the lungs, but it also affects other organs, such as the liver, spleen, and brain. Approaches for selectively inhibiting lung inflammation may provide insight on the systemic effects of O_3_ and could involve direct delivery of substances to the lungs through the trachea prior to O_3_ exposure. Volatile anesthetics such as isoflurane are frequently used for intratracheal instillations and other surgeries [1–3]. However, isoflurane can have anti-inflammatory effects [4, 5], which may be most potent in the respiratory tract since it is the first site of exposure. Injectable anesthetics such as ketamine/xylazine are also used for intratracheal instillations [6], but may also modulate inflammatory responses [7–10]. Currently, little is known about how anesthesia prior to O_3_ exposure affects inflammatory responses. To test this, we exposed mice to isoflurane (Iso) or ketamine/xylazine followed by atipamezole reversal (K/X/A) prior to O_3_. Conditions of anesthesia were designed to replicate those used for non-surgical intratracheal instillations of substances such as drugs or biologics that modulate effects of O_3_ on the lungs in mice. The day after O_3_ exposure, mice were evaluated for markers of systemic and pulmonary inflammation. We found that Iso and K/X/A modify lung inflammation in response to O_3_.

## Methods

### Animal Use

Female CD-1 mice, age 10–12 weeks, (Charles River Laboratories, Malvern, PA, USA) were used for air and O_3_ exposures. Females were used because they have more robust inflammatory responses to O_3_. Mice were given food and water ad libitum and kept on a 12-h light/12-h dark cycle. Protocols were approved by the institutional animal care and use committee of the VA Puget Sound Healthcare System.

### Anesthesia

**Fig. 1:**
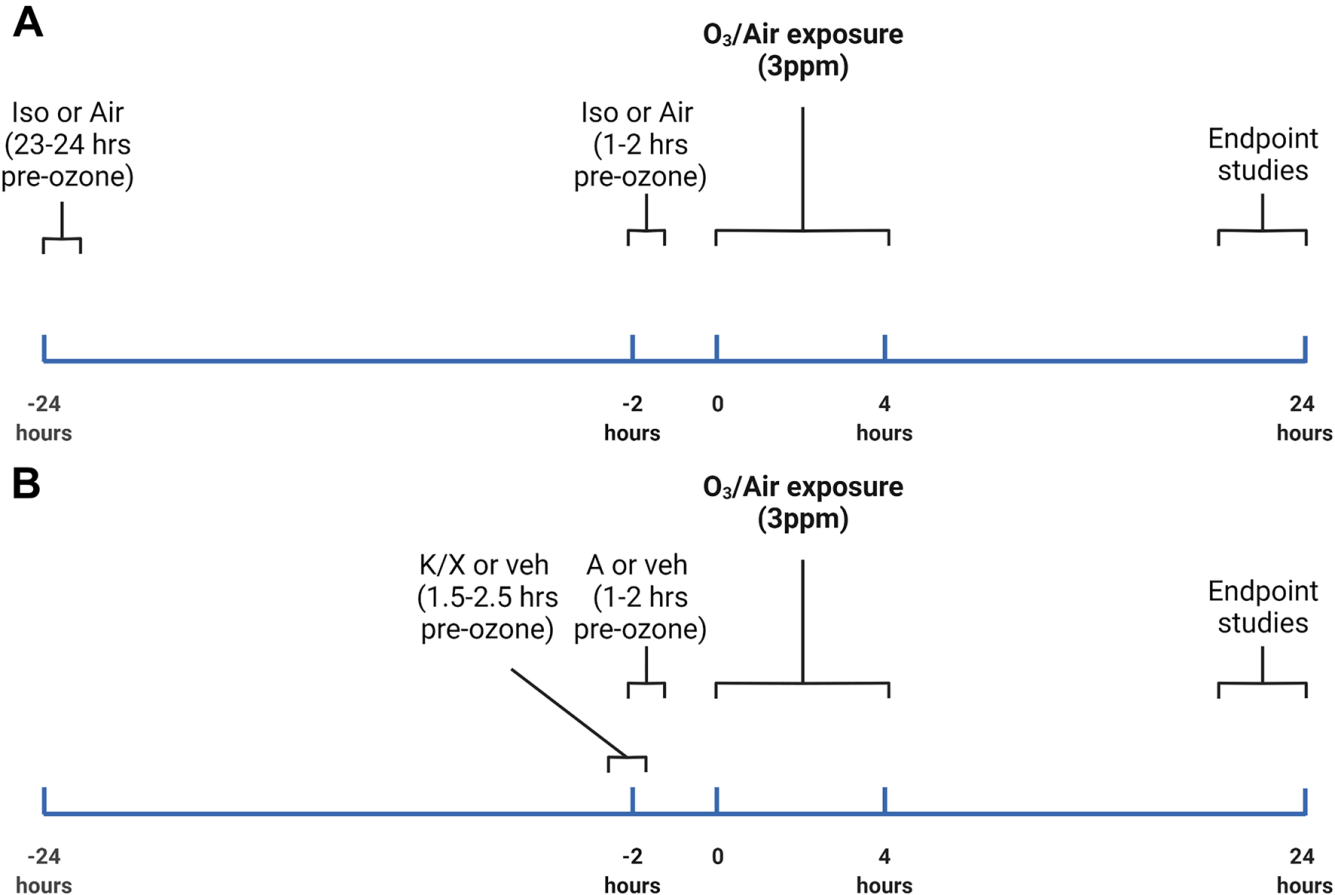
Schematic of anesthesia/ozone (O_3_) exposure protocols. **A** shows the exposure protocol for mice pre-exposed to isoflurane (Iso) or air control, and **B** shows the exposure protocol for mice pre-exposed to Ketamine/Xylazine (K/X) and atipamezole (A) or saline vehicle. Created with Biorender.com

Isoflurane was administered by first weighing all mice, followed by placing mice in an induction chamber filled with 4% isoflurane for 1.5 min and then transferring to a nose cone where 3% isoflurane was used to maintain deep anesthesia for 5 min, which approximates the maximum time that a skilled technician needs to complete an intratracheal injection. Control mice were placed in the empty anesthesia chamber with room air for 1.5 min and then returned to their home cage. 1–2 h or 23–24 h after isoflurane exposure, O_3_ exposures began. Ketamine (80 mg/kg) and xylazine (10 mg/kg) in sterile saline were administered by intraperitoneal injection (IP). 20–30 min after the injection, mice were given 1 mg/kg atipamezole IP to quickly counteract the effects of the anesthesia. Control mice were given IP saline. 1–2 h after the atipamezole injection, O_3_ exposures began. All mice were numbered with a marker to track their identity, and mice were randomized so that the average time between anesthesia and air/ozone exposure was equivalent between groups. A schematic of O_3_ and anesthesia regimens is shown in Fig. 1.

### ***O***_***3***_*** Exposure***

O_3_ exposures were conducted at 3 ppm for 4 h (10:00–14:00), which induce a robust inflammatory response without inducing respiratory distress [11]. For the exposure duration, 3–4 mice were housed in standard mouse cages with free access to water, but without food or bedding to prevent consumption of ozonated food or bedding materials. Up to four cages at a time were placed in a 30″ × 20″ × 20″ polypropylene chamber where O_3_ (3 ppm, chamber 1) or compressed dry air (chamber 2) was pumped into the chamber at equivalent rates. Anesthesia and O_3_ exposures were replicated at least once to capture day-to-day variability, and results for each dose showed consistent trends. O_3_ levels in the chambers were generated and regulated using an Oxycycler AT42 system (BioSpherix, Parish NY, USA). Prior to each experiment, the system was calibrated using a model 106-L O3 detector (2B Technologies, Boulder, CO, USA), and O_3_ levels were recorded from an inlet valve in one of the O_3_-exposed mouse cages every 10 s for the duration of exposures. In all experiments, O_3_ achieved its target concentration within 10 min, and levels were regulated within 10% of the target concentration (3 ppm ± 0.3 ppm) thereafter.

### Blood Collection and Serum Amyloid A Measurement

22–24 h after the start of O_3_ exposure (12:00–14:00), mice were re-weighed and deeply anesthetized with IP urethane (Millipore Sigma, St. Louis, MO, USA). Blood was collected from the abdominal aorta, allowed to clot for 30 min, and then placed on ice. Blood was centrifuged at 2500×*g* for 15 min and serum was collected, aliquoted, and frozen at − 80 °C. Serum was diluted 1/10,000 for mice exposed to ozone and 1/50 for mice not exposed to ozone. Serum amyloid A (SAA) mouse duoset kits (R and D systems, Minneapolis, MN, USA) were used to quantify SAA in serum.

### WLL and Pulmonary Inflammation Assessment

Whole-lung lavage (WLL) was performed on mice by perfusing and aspirating the lungs through the trachea three times with 1 mL sterile phosphate-buffered saline (3 mL total). WLL fluid was stored on ice and centrifuged at 200×*g* for 5 min at 4 °C. The supernatant was removed and 0.5 mLs of supernatant were reserved to resuspend the cell pellet. The remainder was frozen for measurement of total WLLL protein, which was performed using a microBradford assay. Total cells were manually counted using a hemacytometer. Differential cell counts were performed on Hemacolor-stained cytocentrifuge preparations. Cell counts were performed using ImageJ, and at least 200 cells were counted to determine relative cell proportions.

### Statistics

Statistical analysis was done with Prism 8.4.2 (GraphPad Software, San Diego, CA, USA). Data are reported as mean ± SD and analyzed by two-way ANOVA for main effects and interactions and Tukey’s multiple comparisons test for comparisons of group means.

## Results

### Effects of Isoflurane and Ketamine/Xylazine/Atipamezole Anesthesia on Ozone-Induced Pulmonary Inflammation and Injury

**Fig. 2:**
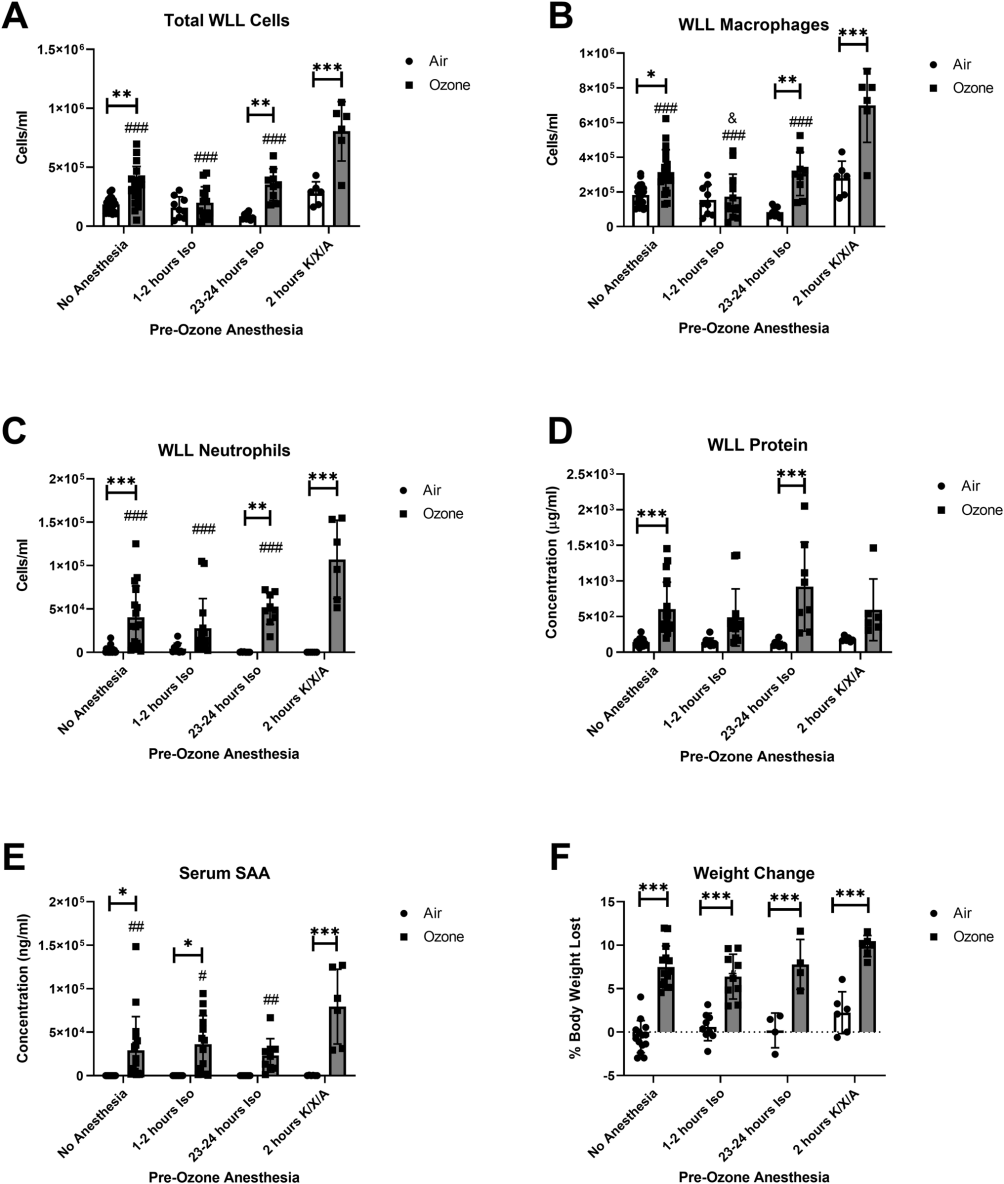
Effects of isoflurane (Iso) and ketamine/xylazine/atipamezole (K/X/A) on inflammatory (**A**–**C**) and vascular damage (**D**) markers in the lung, blood levels of serum amyloid A (SAA, **E**), and weight loss (**F**). In all panels, *n* = 6–20 per group. ***p < 0.001, **p < 0.01, *p < 0.05, air vs. O3. ^###^*p* < 0.001, ^##^*p* < 0.01, ^#^*p* < 0.05, vs. K/X/A + O_3_ group. ^&^*p* < 0.05 vs. No Anesthesia + O_3_. Group mean comparisons were carried out using Tukey’s multiple comparisons test

We first determined whether O_3_-induced responses in the lungs differed with Iso or K/X/A. Since drugs or other interventions to be administered intratracheally would ideally be given a short time before exposure to ozone to maximize their effects/minimize their metabolism, we evaluated anesthesia administration at 1–2 h prior to ozone exposure. We chose 1–2 h because this time frame allows the mice to recover from anesthesia and also reflects a time window that is typically sufficient for an administered drug to sufficiently activate/inhibit its biological target. A pilot study indicated that Iso given 1–2 h before O_3_ inhibited inflammation, suggesting that this regimen is unsuitable for studying anti-inflammatory interventions for O_3_ exposure. Therefore, we included a 24-h Iso pre-exposure in experimental replicates to determine whether the anti-inflammatory effects of Iso were sustained. The 24-h time point was chosen because the Iso and O_3_ exposures could then occur at approximately the same time of day as the 1–2-h Iso pre-exposure group. We compared K/X/A to Iso 1–2-h pre-O_3_ exposure to determine whether K/X/A is a suitable alternative anesthesia regimen to Iso (Schematics in Fig. 1A, B). Although the controls for ketamine were IP injected with saline, whereas Iso controls were not, we found that IP injections did not significantly influence the measured outcomes, and so we combined the Iso and Ketamine no anesthesia control groups for analysis. O_3_ significantly increased WLL total cells, macrophages, and neutrophils except in the 1–2-h Iso group (Fig. 2A–C). We further show that mice exposed to K/X/A and O_3_ had significantly increased WLL total cells, macrophages, and neutrophils vs. other O_3_-exposed groups. O_3_ significantly increased WLL protein in the no anesthesia group and 23–24-h Iso groups, but not in the 1–2-h Iso or K/X/A groups (Fig. 2D). The *F*-statistics and *p*-values for main effects and interactions are reported in Table 1.

**Table 1:** Two-way ANOVA analysis results showing percentages of variation by factor (main effects, interactions) and *F*-statistics

Figure	Anesthesia	Exposure	Interaction
2A—total WLL cells	28.97%; *F* (3,82) = 22.15, *p* < 0.0001	29.96%; *F* (1,82) = 68.73, *p* < 0.0001	12.79%; *F* (3,82) = 9.780, *p* < 0.0001
2B—WLL macrophages	31.77%; *F* (3,82) = 23.49, *p* < 0.0001	25.59%; *F* (1,82) = 56.74, *p* < 0.0001	11.72%; *F* (3,82) = 8.662, *p* < 0.0001
2C—WLL neutrophils	9.766%, *F* (3,82) = 6.535, *p* = 0.0005	44.88%; *F* (1,82) = 90.08, *p* < 0.0001	11.82%; *F* (3,82) = 7.907, *p* = 0.0001
2D—WLL total protein	2.776%; *F* (3, 81) = 1.309, *p* = 0.2773	33.06%; *F* (1, 81) = 46.76, *p* < 0.0001	3.888%; *F* (3,81) = 1.833, *p* = 0.1478
2E—serum SAA	7.296%, *F* (3,81) = 3.668, *p* = 0.0156	35.35%; *F* (1, 81) = 53.31, *p* < 0.0001	7.204%; *F* (3,81) = 3.622, *p* = 0.0165
2F—weight change	7.784%; *F* (3, 58) = 6.412, *p* = 0.0008	52.74%; *F* (1, 58) = 130.3, *p* < 0.0001	1.903%; *F* (3,58) = 1.567, *p* = 0.2070

For Fig. 2D, one sample was excluded because of apparent blood contamination. For Fig. 2E, one sample was excluded because not enough serum was recovered to complete the assay. For Fig. 2F, body weights were not measured in one experimental replicate and so 23 samples are missing

### Effects of Iso and K/X/A Anesthesia on Ozone-Induced Systemic Inflammation and Weight Loss

We next determined whether the effects of anesthesia extended to markers of ozone-induced systemic inflammation and behavioral effects. We have previously shown that the acute-phase protein SAA is consistently increased in blood following a 3 ppm exposure to O_3_ and its upregulation occurs in the absence of changes in other pro-inflammatory cytokines in blood [12]. Weight loss is a measurable outcome of sickness behavior, which can be induced by O_3_. Anesthesia has no effect on O_3_-induced weight loss based on the post hoc mean comparisons of the ozone groups (Fig. 2F); however, there was a significant main effect of treatment that explained 5.172% of the total variation (Table 1). O_3_ significantly increased SAA levels in all groups except for mice exposed to Iso 23–24 h prior (Fig. 2E). SAA levels induced by O_3_ were significantly higher in mice pre-anesthetized with K/X/A vs no anesthesia or Iso.

## Discussion

We found that isoflurane inhibits O_3_-induced pulmonary inflammation if given 1–2 h prior to exposure, and this effect diminishes by 24 h. Isoflurane induces anesthesia via potentiation of inhibitory γ-aminobutyric acid type A (GABA A) receptors and inhibition of glutamatergic α-amino-3-hydroxy-5-methyl-4-isoxazolepropionic acid (AMPA) and *N*-methyl-d-aspartate (NMDA) receptors [13]. Isoflurane also has anti-inflammatory activities in a variety of rodent models [4], although the mechanisms are not well defined. It has been shown that activation of GABA A receptors and inhibition of NMDA receptors have anti-inflammatory effects on immune cells [14–16], suggesting that the anti-inflammatory activities of Iso could be mediated through direct actions on leukocytes. In contrast to Iso, K/X/A increased O_3_-induced inflammatory responses to O_3_. Anti-inflammatory/protective effects through NMDA receptor antagonism have been described for ketamine and ketamine/xylazine combinations [9]. Whereas xylazine is a α2-adrenergic receptor agonist and potentiates anesthesia, atipamezole is an antagonist and leads to rapid anesthesia reversal within about 20 min [17]. The data on pro- vs. anti-inflammatory activities of the α2 adrenergic receptor are equivocal; however, a recent study showed that atipamezole may promote or prolong inflammation through its action via a α2-independent mechanism [18]. It is also possible that anesthetics affect the amount of leukocyte trafficking to the lungs by altering the cytokine and chemokine profiles that are induced by O_3_. Acute exposures to O_3_ upregulate cytokines and chemokines such as CCL11, IL-6, G-CSF, CXCL10, and CXCL1 in a dose-dependent and cell type-specific manner [19]. Cytokines, such as TNF-α, IL-1, and IL-6, chemokines, such as CXCL1, CXCL10, and CCL7, and chemokine receptors such as CCR2 have been shown to contribute to leukocyte trafficking to the lungs in response to O_3_ [20–25]. Future work is needed to delineate the immunomodulatory mechanisms of Iso and K/X/A.

Our results are the first to show that isoflurane exposure prior to O_3_ inhibits pulmonary inflammation, whereas K/X/A exposure has an enhancing effect. These results complement other recent works that have investigated anesthesia’s effects on pulmonary inflammation in response to ozone and other pro-inflammatory agents [5, 16, 26] and effects of anesthetics on the recovery of cells from WLL [27]. Although the scope of our study is limited in that we did not define a full-time course of anesthesia’s effects, our results highlight that brief, acute anesthetic regimens can alter inflammatory responses to O_3_. We conclude that anesthetics are important experimental variables whose evaluation is necessary for studies requiring their use prior to exposure to O_3_ or other pathological stimuli.
